# Real-Time Navigation in Liver Surgery Through Indocyanine Green Fluorescence: An Updated Analysis of Worldwide Protocols and Applications

**DOI:** 10.3390/cancers17050872

**Published:** 2025-03-03

**Authors:** Pasquale Avella, Salvatore Spiezia, Marco Rotondo, Micaela Cappuccio, Andrea Scacchi, Giustiniano Inglese, Germano Guerra, Maria Chiara Brunese, Paolo Bianco, Giuseppe Amedeo Tedesco, Graziano Ceccarelli, Aldo Rocca

**Affiliations:** 1 Department of Clinical Medicine and Surgery, University of Naples “Federico II”, 80138 Naples, Italy; 2 Hepatobiliary and Pancreatic Surgery Unit, Department of General Surgery, Pineta Grande Hospital, 81030 Castel Volturno, Italy; 3 Department of Medicine and Health Science “V. Tiberio”, University of Molise, 86100 Campobasso, Italy; 4 School of Medicine and Surgery, University of Milano-Bicocca, 20126 Monza, Italy; 5 Division of General and Minimally Invasive Surgery, Department of Surgery, San Giovanni Battista Hospital, 06034 Foligno, Italy; 6 Minimally Invasive and Robotic Surgery Unit, San Matteo Hospital, 06049 Spoleto, Italy

**Keywords:** indocyanine green (ICG) fluorescence, liver surgery, tumor detection, segmentation, fluorescence-guided surgery

## Abstract

Background: Indocyanine green (ICG) fluorescence has seen extensive application across medical and surgical fields, praised for its real-time navigation capabilities and low toxicity. Initially employed to assess liver function, ICG fluorescence is now integral to liver surgery, aiding in tumor detection, liver segmentation, and the visualization of bile leaks. This study reviews current protocols and ICG fluorescence applications in liver surgery, with a focus on optimizing timing and dosage based on clinical indications. Methods: Following PRISMA guidelines, we systematically reviewed the literature up to 27 January 2024, using PubMed and Medline to identify studies on ICG fluorescence used in liver surgery. A systematic review was performed to evaluate dosage and timing protocols for ICG administration. Results: Of 1093 initial articles, 140 studies, covering a total of 3739 patients, were included. The studies primarily addressed tumor detection (40%), liver segmentation (34.6%), and both (21.4%). The most common ICG fluorescence dose for tumor detection was 0.5 mg/kg, with administration occurring from days to weeks pre-surgery. Various near-infrared (NIR) camera systems were utilized, with the PINPOINT system most frequently cited. Tumor detection rates averaged 87.4%, with a 10.5% false-positive rate. Additional applications include the detection of bile leaks, lymph nodes, and vascular and biliary structures. Conclusions: ICG fluorescence imaging has emerged as a valuable tool in liver surgery, enhancing real-time navigation and improving clinical outcomes. Standardizing protocols could further enhance ICG fluorescence efficacy and reliability, benefitting patient care in hepatic surgeries.

## Simple Summary

Indocyanine green (ICG) fluorescence has become a crucial tool in liver surgery due to its real-time navigation capabilities and low toxicity. Initially used for liver function assessment, it now plays a significant role in tumor detection, liver segmentation, and bile leak visualization. This systematic review, conducted following PRISMA guidelines, analyzed 140 studies comprising 3739 patients to evaluate ICG dosage and timing protocols. The most common dosage for tumor detection was 0.5 mg/kg, administered days to weeks before surgery. Tumor detection rates averaged 87.4%, with a 10.5% false-positive rate. Various near-infrared (NIR) camera systems were used, with the PINPOINT system being the most frequently cited. Other applications included bile leak detection, lymph node mapping, and vascular and biliary structure visualization. The findings highlight ICG fluorescence as a valuable tool in liver surgery, with the standardization of protocols potentially enhancing its efficacy and reliability for improved patient outcomes.

## 1. Background

Over the past few decades, indocyanine green (ICG) fluorescence has greatly evolved and has achieved extraordinary diffusion in many medical and surgical fields [1,2,3,4,5,6,7]. ICG is a water-soluble fluorescent dye that is metabolized by the liver and extracted through bile ducts, with a half-life of three minutes after systematic injection [8]. Furthermore, it is minimally toxic (nausea and pyrexia were rarely reported) and it represents a suitable contrast agent for real-time navigation due to its 820 nm near-infrared (NIR) emission wavelength, minimizing interference from blood and tissue autofluorescence at 500–600 nm [8].

From early ICG application in 1950, it was largely used to assess liver function and perform surgical strategies based on Future Remnants Liver (FRL) and in other abdominal surgeries to identify tumors and lymphatic mapping and to evaluate organ and anastomosis perfusions [9].

In 2008, Aoki et al. [1] described the first application of ICG fluorescence through its intraoperative portal injection to identify hepatic segments. In 2013, Ishazawa et al. [10] definitively showcased the utility of ICG fluorescence when administered during the preoperative period. Starting with those publications, the effectiveness and safety of ICG have been further reported by many authors [4,7,11,12,13,14] who have enlightened its value in detecting tumors, either on liver surfaces or surgical specimens. Thanks to its broad availability and cost-effectiveness, ICG has been an increasing diffusion [15].

In the last 10 years, due to the widespread of robotic platforms [16,17,18,19,20,21,22,23,24] and the improvement in laparoscopic optics, ICG fluorescence has also been widely adopted in Western countries, especially in liver and colorectal surgery [8,25,26,27,28,29,30].

However, many limitations were described: the depth of liver tissue detectable is approximately less than 10 mm [31], and in patients with a poor ICG fluorescence retention rate (i.e., decreased clearance function of the liver), the fluorescence signal of the non-cancerous liver parenchyma was higher [32]. These findings impact the adequate tumor contrast, especially when ICG fluorescence was administered to patients affected by cirrhosis or steatosis or those who had undergone neoadjuvant chemotherapy.

Hence, ICG clinical application is still not well standardized. Consequently, our purpose was to examine the current evidence and explore the practical uses of ICG fluorescence for tumor detection, hepatic segmentation, bile leak identification, and lymph nodes mapping, carrying out a literature review of the timing of administration and dosage in several liver surgery applications and diseases.

## 2. Material and Methods

### 2.1. Literature Review

Our systematic review included articles published until 27 January 2024, following Preferred Reporting Items for Systematic Reviews and Meta-Analyses (PRISMA) guidelines [33] and Assessing the methodological quality of Systematic Reviews (AMSTAR) [34].

This systematic review has been registered in the International Prospective Register for Systematic Reviews—PROSPERO, with registration number CRD42024581500.

We conducted a comprehensive search of PubMed and Medline databases to find original English papers focusing on ICG fluorescence uses in clinical studies related to liver surgery. Studies were selected using the following medical subject headings (MeSH): ((ICG fluorescence [Title]) OR (indocyanine green [Title])) AND (((liver) OR (liver surgery) OR (liver resection [Title]) OR (liver transplantation [Title]))). Furthermore, the search strategy was flexibly modified based on the specific database requirements, and further expanded by thoroughly identifying potential references from the screened texts. All authors discussed and approved the search strategy.

All anatomic, cadaveric, and surgical studies on ICG fluorescence in liver surgery were analyzed. An early exclusion was performed based on titles and abstracts by three authors (P.A., S.S., and M.R.). Furthermore, duplicates were left out. At least two blinded authors (S.S. and M.R.) used Rayyan to identify and analyze relevant studies [35]. Disagreements on article selection were resolved by discussion and consensus among the authors. All reports obtained from the databases will be exported to the reference management EndNote software (EndNote™ 21 version, Clarivate, Philadelphia, PA, USA). The duplicated reports were managed according to the Bramer et al. method [36]. The final review of the articles was carried out in April 2024.

### 2.2. Inclusion Criteria

The main study objectives have been formulated according to the Patients, Intervention, Comparison, and Outcomes method [37] (Table 1). English language, human studies, and full-text available studies were included only if the dose and timing of ICG fluorescence administration were clearly described. Comparative studies, case series, and case reports that met these inclusion criteria were involved. All benign and malignant liver neoplasms were collected (Hepatocellular Carcinoma (HCC), Cholangiocarcinoma (CCA), Colorectal Liver Metastases (CRLM), Primary Liver Cancer (PLC), Focal Nodular Hyperplasia (FNH), Gastrointestinal Stromal Tumor (GIST), Squamous Cell Carcinoma (SCC), and Liver Transplant (LT)).

### 2.3. Exclusion Criteria

Studies involving animal experiments, studies published in languages other than English, conference abstracts, editorials, expert opinions, and review papers were excluded. Patients’ sample size, age, and surgical approaches (open, laparoscopic, and/or robotic surgery) do not represent exclusion criteria.

### 2.4. Data Extraction and Endpoints

Reports were divided into three distinct groups, namely irrelevant, relevant, and unsure. Studies determined to be irrelevant by both reviewers were excluded, while papers that were relevant or unsure by at least one reviewer were analyzed thanks to a full-text evaluation.

**Figure 1:**
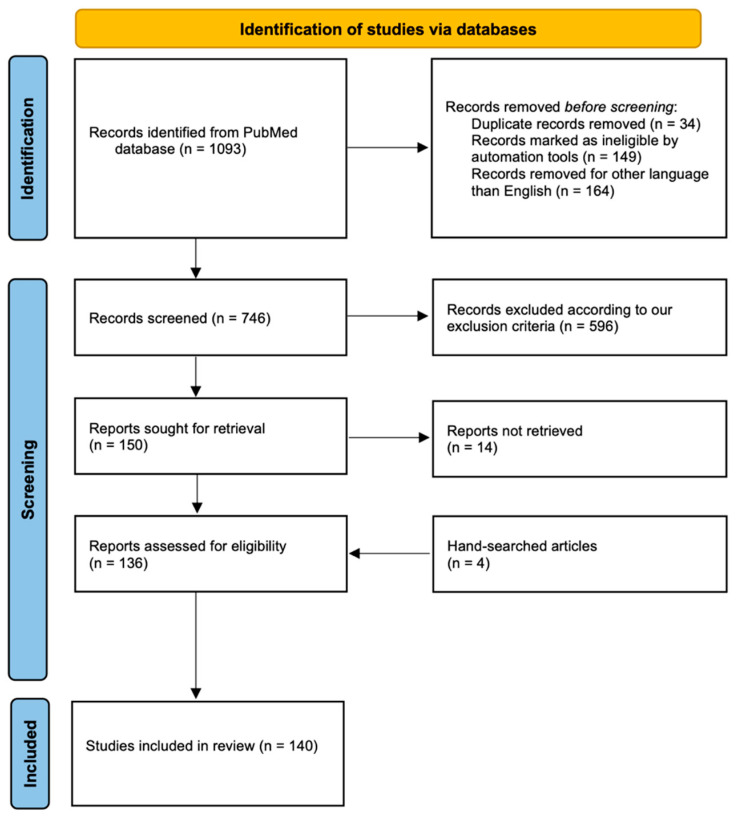
PRISMA Flowchart of our systematic analysis.

A PRISMA flow chart documented the entire search and selection process (Figure 1).

Using a standardized data extraction form, all authors recorded key details for each study, such as the first author, year of publication, study design, total number of patients, type of surgery (open, laparoscopy, or robotic surgery), type of laparoscopic/robotic system, type of NIR, liver disease, age, percentage of patients affected by cirrhosis, ICG fluorescence dose (mg/kg or mL), timing of administration, type of navigation (tumor detection, segmentation or other uses), and percentage of major hepatectomies (≥3 liver segments resection). In the case of missing information, the authors performed calculations based on the available data.

Data were divided into six tables (Tables S1–S6) according to different ICG fluorescence uses.

The primary endpoint was to evaluate the dosing and timing of ICG fluorescence in liver surgery in tumor detection. In order to achieve data about liver segmentation, we analyzed the direct portal ICG injection under percutaneous-guided ultrasound, defined as the “*positive staining*” technique or the “*negative staining*” technique obtained through an intravenous ICG dye injection after segmental portal pedicle clamp, following the Glissonian approach [38].

Our secondary endpoint was to collect data regarding other possible ICG fluorescence applications in clinical practices, such as bile leak, bile duct visualization, hepatic artery embolization, and lymph node sentinel.

### 2.5. Ethical Issues

Ethical approval or informed consent is not required since this systematic review will be based only on previously published studies and does not imply any direct contact with individual subjects.

## 3. Results

A total of 1093 articles were initially obtained (Figure 1). According to exclusion criteria, 957 studies were excluded because they were not relevant or duplicates. After the addition of 4 hand-searched studies, 140 articles were finally selected [1,6,10,12,13,14,26,27,28,31,39,40,41,42,43,44,45,46,47,48,49,50,51,52,53,54,55,56,57,58,59,60,61,62,63,64,65,66,67,68,69,70,71,72,73,74,75,76,77,78,79,80,81,82,83,84,85,86,87,88,89,90,91,92,93,94,95,96,97,98,99,100,101,102,103,104,105,106,107,108,109,110,111,112,113,114,115,116,117,118,119,120,121,122,123,124,125,126,127,128,129,130,131,132,133,134,135,136,137,138,139,140,141,142,143,144,145,146,147,148,149,150,151,152,153,154,155,156,157,158,159,160,161,162,163,164,165,166,167,168]: 2 comparative studies, 1 case series, 26 case reports, 1 case-matched study, 1 cohort study, 75 retrospective studies, 6 Randomized Clinical Trials (RCTs), 18 prospective studies, 1 post hoc analysis of a prospective study, and 9 types of study not specified. The cumulative study population encompassed 3739 patients. The publication period ranges from 2009 to 2024. The PRISMA flow diagram outlining the study selection process is presented in Figure 1.

**Figure 2:**
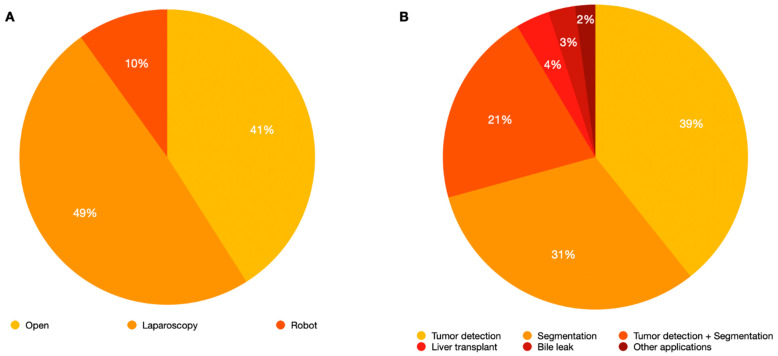
(**A**) Percentage of open, laparoscopic, and robotic surgery involved in our review. In 1 report, the type of surgery was not specified; (**B**) Percentage of clinical applications detected.

Several types of NIR cameras were used by authors: the PINPOINT system/1588 Advanced Imaging Modalities Platform (Stryker Co., Kalamazoo, MI, USA) was the most frequent available system (45 studies, 31.9%), followed by Photodynamic Eye (Hamamatsu Photonics Co., Shizuoka, Japan) (24 reports, 17%). The Firefly system (Intuitive Surgical Inc., Sunnyvale, CA, USA) was used in 16 articles (11.3%) for robotic surgery. Nevertheless, other NIR types were not available in 56 (39.7%) studies (Tables S1–S6). Several clinical applications of ICG fluorescence were reported: 56 articles (40%) described tumor detection ability, 47 articles (33.6%) described liver segmentation ability, and in 30 (21.4%) articles, the authors experienced both applications. Limited experiences are reported in bile leak detection five (3.6%) articles. Seven articles (5%) reported other uses. The type of surgery (open vs. minimally invasive surgery) and clinical application of ICG fluorescence are summarized in Figure 2. As reported in Tables S1–S6, cirrhotic patients were included in many studies, with a percentage of liver cirrhosis with available data of 47.8%.

ICG fluorescence was administered preoperatively, intraoperatively, and in both periods. The dose most frequently used was 0.5 mg/kg (Tables S1–S6). Generally, ICG fluorescence was administered within 14 days before the operation day, especially within 3 days in many of the studies. An additional administration (0.02–0.5 mg/kg) was described as an option in case of the long interval between the administration and operation day. Finally, the median tumor detection rate was reported to be 87.4% (43–100%), and the median false-positive rate was reported to be 10.5% (0–31.3%).

The negative staining method was reported in 55 (39%) articles. Most authors performed this technique using 2.5 mg/body (range 0.025–25 mg/body) intravenously. Conversely, 35 (24.8%) articles carried out the positive staining approach, frequently using an ICG fluorescence dose of 0.25 mg/body (range 0.025–12.5 mg/body).

Eight studies reported an innovative application of ICG fluorescence during real-time liver navigation. Overall, 5 (3.6%) out of 140 articles described intraoperative injection of ICG fluorescence to detect bile leaks, either in open or minimally invasive liver surgery (Table S5).

### 3.1. ICG Fluorescence Imaging for HCC Detection

**Figure 3:**
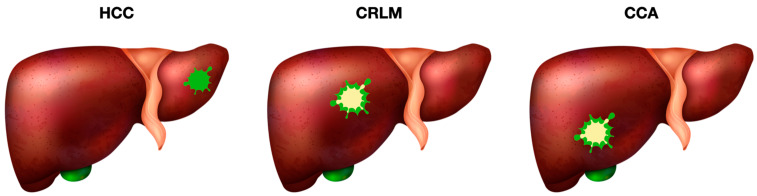
Indocyanine green fluorescence imaging in HCC, CRLM, and CCA during liver surgery. Abbreviations: HCC, Hepatocellular Carcinoma; CRLM, Colorectal Liver Metastases; CCA, Cholangiocellular Carcinoma.

Generally, HCC lesions exhibit total tumor fluorescence upon superficial examination (Figure 3). However, discrepancies were noted when comparing surface observations with sectional views. Cases where surface and sectional fluorescence patterns were consistent predominantly involved tumors exposed on the hepatic surface. Contrarily, tumors located deeper within the liver showed total fluorescence superficially but partial or rim fluorescence upon sectioning, suggesting that tumor depth influences ICG fluorescence imaging clarity.

Nevertheless, poorly differentiated HCCs showed variations in fluorescence patterns (partial or rim-type), maybe due to slower ICG excretion in less differentiated cancer cells.

### 3.2. ICG Fluorescence for CRLM Detection

In CRLM, ICG fluorescence imaging shows characteristic rim fluorescence patterns: fluorescence is primarily observed in non-tumor cells at the periphery, while tumor cells in the central region remain non-fluorescent (Figure 3).

Superficially, CRLM lesions often exhibited total fluorescence, but discrepancies emerged when comparing surface and sectional views. Tumors located closer to the hepatic surface generally displayed consistent fluorescence patterns between surface and cross-sectional imaging, whereas deeper lesions appeared fluorescent superficially but exhibited partial or rim fluorescence upon sectioning. Additionally, larger and more fibrotic CRLM lesions tended to show greater heterogeneity in fluorescence distribution, possibly due to altered vascularization and ICG uptake dynamics in metastatic tissue.

### 3.3. ICG Fluorescence for CCA Detection

In CCA, ICG fluorescence imaging typically revealed a rim fluorescence pattern, with fluorescence primarily observed in non-tumor cells surrounding the lesion, while tumor cells in the central region remained non-fluorescent (Figure 3). This peripheral fluorescence is likely due to ICG uptake by peritumoral hepatocytes and retention in fibrotic stromal components rather than direct tumor cell accumulation.

Superficially, as reported for CRLM, CCA lesions often appeared fluorescent, but inconsistencies arose when comparing surface and sectional views. Additionally, poorly differentiated ICC and tumors with extensive fibrosis exhibited greater heterogeneity in fluorescence patterns, possibly due to reduced ICG penetration and altered vascular permeability within the tumor microenvironment.

## 4. Discussion

In the last decades, ICG fluorescence has been used to assess the hepatic and choroidal blood flow and the measurement of cardiac output [3,4,9]. The main advantage of ICG fluorescence imaging is its non-invasive nature and real-time capability and it does not require direct manipulation of vessels or prolonged imaging times, reducing intraoperative time and potential complications associated with invasive techniques. Furthermore, the safety profile of ICG fluorescence is well-established, with minimal allergic reactions or adverse effects reported in clinical practice.

Nevertheless, the standardization of dosage and timing of administration still represent crucial issues in a challenging scenario such as Hepato-pancreatic and Biliary (HPB) surgery. It could be linked to several preferred dosages and timing and the lack of literature data due to the heterogeneity of populations, devices, and tumor features. Furthermore, age and liver texture could impact intraoperative ICG fluorescent emission and visualization. Based on these findings, a consensus statement on ICG fluorescence used in liver surgery is a key prospect for the future.

Nevertheless, the European Association for Endoscopic Surgery (EAES) published a consensus conference with several statements for ICG application in abdominal surgery, including HPB [9].

**Figure 4:**
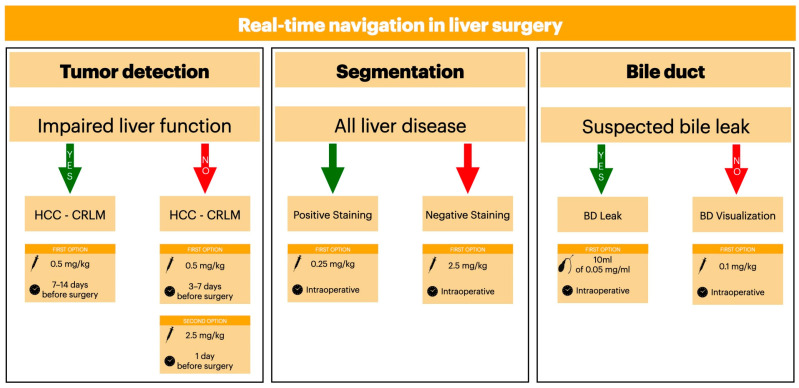
The flowchart of ICG doses and timing according to our systematic review. The syringe indicates intravenous administration, while the biliary tree implies trans-biliary duct administration. No protocols were reported for cholangiocarcinoma detection due to limited data availability. Abbreviations: HCC, Hepatocellular Carcinoma; CRLM, Colorectal Liver Metastases; BD, Bile Duct.

Given the well-established effectiveness of ICG fluorescence in improving clinical outcomes [80,124,131,145,150], our review offers a flowchart of ICG fluorescence application based on the most used dosages and timing for hepatic neoplasm identification, liver segmentation, and bile leak visualization during open and minimally invasive surgery (Figure 4).

### 4.1. Tumor Detection

The application of ICG fluorescence imaging in HPB surgery has been widely explored, demonstrating utility in tumor detection and the dose and timing of its administration differ significantly across studies. The lack of ICG fluorescence in healthy tissue represents a real-time guide for the dissection plane, allowing for higher R0 resection rates. A common dosing strategy involves administering 0.5 mg/kg of ICG fluorescence preoperatively.

The previous studies by Gotoh et al. [6] and Ishizawa et al. [13] reported a clinical experience in a limited number of patients (10 and 26 patients, respectively) affected by HCC using a 0.5 mg/kg dose with a timing range of 1–8 days before open liver surgery. Contrarily, Uchiyama et al. [39] and Peloso et al. [41] performed liver resection after administration of 0.5 mg/kg of ICG fluorescence with a timing of 2 weeks and 24 h before surgery, respectively, in open surgeries for CRLM.

Following these early experiences, several studies explored different doses and timings to optimize detection (Table S1). Van der Vorst et al. [44] conducted a Randomized Clinical Trial (RCT) involving 40 patients and compared 10 mg and 20 mg doses administered 24 or 48 h before surgery for CRLM detection, highlighting that variations in dosing and timing can be tailored based on specific clinical needs and tumor types.

As reported in Table S1, ICG fluorescence imaging has been employed in both open and minimally invasive surgeries, demonstrating its versatility. The retrospective study by Kudo et al. [45] utilized ICG fluorescence in minimally invasive surgeries for HCC and CRLM, with a dose of 0.5 mg/kg administered within 2 weeks preoperatively.

An interesting application was reported by Tummers et al. [47] who used ICG in a case report involving minimally invasive surgeries for melanoma, using a 10 mg dose administered 1 day before surgery.

As reported in recent consensus on ICG fluorescence-guided surgery by Casinotti et al. [9], ICG fluorescence aids in identifying subcapsular and superficial tumors, as well as deeper lesions, and in locating unknown glissonian tumors and ensuring anatomical resection margins. Considering these findings, several studies compared ICG fluorescence application to Intra-Operative UltraSound (IOUS) [145].

While IOUS is widely considered the gold standard for tumor detection, ICG use has demonstrated superior accuracy in identifying lesions smaller than 2 mm [26]. IOUS and ICG combination increase the sensitivity in locating superficial lesions [6,28].

Nevertheless, in cirrhotic and hardened liver ICG fluorescence can be especially beneficial due to difficult IOUS interpretation and untrustworthy tactile feedback. In addition, in this group, the interval between ICG fluorescence administration and surgery is longer due to a higher ICG fluorescence retention rate when compared to healthy livers (Figure 4).

Many studies demonstrated the different penetration of ICG fluorescence through liver tissue: total or partial fluorescence staining pattern is generally due to HCC, while rim fluorescence staining pattern is detected mainly in metastases. Different characteristics of penetration are described for CCA due to there being no predominant staining pattern.

Although Handgraaf et al. [55] and Zhou et al. [138] hold up the false positive in tumor detection due to the non-specific uptake of lesions with consequent benign lesion resections, similar findings are reported by Nishino et al. [102] who emphasized the need for careful intraoperative assessment.

Frantz et al. [26] also retrieved a peritoneal metastasis in one patient affected by HCC. The ICG fluorescence dose was similar to other patients included in their study. No other superimposable experiences were found in the literature review.

### 4.2. Segmentation

The application of ICG fluorescence imaging for liver segmentation during HPB surgery has been the subject of numerous studies, as listed in Table S2.

ICG fluorescence imaging has shown significant efficacy in intraoperative liver segmentation, and it is crucial for precise surgical interventions. The dosing and timing of ICG fluorescence administration vary considerably across studies. Timing also varied, with most authors opting for intraoperative administration (Figure 4).

Aoki et al. [1], involving 35 patients undergoing open surgery for various liver diseases, demonstrated effective segmentation using 1 mL of ICG fluorescence (5 mg/mL) administered intraoperatively. In addition, Aoki et al. described the first application of direct portal injection of ICG fluorescence [1]. Furthermore, following previous experience, Aoki et al. [90] used 5 mg of intraoperative ICG fluorescence to detect positive staining in 81 patients affected by primary and secondary liver tumors who underwent minimally invasive surgery.

A lower dose was described by Uchiyama et al. [91] and Cheung et al. [135]: the authors reported successful segmentation in patients affected by HCC using an intraoperative dose of 0.5 mg/kg and 0.025 mg/kg, respectively.

Li et al. [140] performed an unusual application of ICG fluorescence: this retrospective study reported a dose of 2.5 mg intraoperatively administered for liver echinococcosis for negative staining.

Regarding negative staining, Wakabayashi et al. performed an extensive review [169] on ICG fluorescence use and they supposed that this intraoperative technique was preferred during hemi-hepatectomy, sectionectomy, and anterolateral segmentectomies, preferably using Takasaki’s extrahepatic Glissonean approach.

On the contrary, ICG-positive staining required the direct injection of ICG fluorescence into the portal branches responsible for resected territories under IOUS guidance with an 18- to 22-gauge spinal or percutaneous transhepatic cholangiodrainage needle. The injection is performed slowly to avoid ICG fluorescence retrograde flow into neighboring segments with undesired staining without hepatic artery clamping. In addition, it is necessary to assess the hepatic segment volume to be resected. For these reasons, positive staining requires IOUS skills.

### 4.3. Tumor Detection and Segmentation

ICG fluorescence imaging has demonstrated substantial efficacy in contemporary tumor detection and liver segmentation. The comparative study by Abo et al. [28] involving 117 patients undergoing open surgery for primary liver cancer (PLC) and CRLM showed that ICG fluorescence was effective when administered preoperatively (0.5 mg/kg) and intraoperatively (1.25 mg). The study reported effective tumor detection and segmentation, which was consistent with findings from other studies such as Terasawa et al. [133] and Zhang et al. [134], who applied similar protocols in minimally invasive surgeries.

For instance, Peyrat et al. [27] administered 0.25 mg/kg preoperatively one day prior, followed by an intraoperative dose ranging from 0.016 to 1.25 mg, achieving effective tumor detection and segmentation. Similarly, Cheung et al. [135] used a preoperative dose of 0.5 mg/kg administered 10 days prior and an intraoperative dose of 0.025 mg for segmentation and tumor detection in minimally invasive surgeries.

These variations reflect attempts to tailor ICG fluorescence usage to specific surgical contexts.

However, Zhang et al. [143] employed an RCT to compare different doses and timings, finding that preoperative administration 24–72 h before surgery combined with intraoperative doses provided optimal results.

### 4.4. Bile Leak Detection

ICG fluorescence imaging for the detection and management of bile leaks during HPB surgery has shown promising results, as reflected in various studies summarized in Table S5. Our discussion integrates these findings to highlight the effectiveness, application variations, and limitations of ICG fluorescence in preventing and managing bile leaks.

Sakaguchi et al. [161] conducted a cohort study with 27 patients undergoing open surgery for HCC, CRLM, and other liver diseases, utilizing a dose of 0.05 mg/mL of ICG administered intraoperatively. In detail, for the bile leak test, ICG fluorescence was slowly injected into the bile duct through a transcystic tube. The distal common bile duct was clamped. Before injection, the bile reflux using the aspiration technique was confirmed. Bile leaks were easily detected by the extra-biliary fluorescent signal using an infrared camera system (PDE; Hamamatsu Photonics KK, Hamamatsu, Japan).

Similarly, Kaibori et al. [162], in an RCT involving 102 patients, compared an ICG fluorescence control group with an ICG fluorescence PDE group, using 10 mL of a dilute ICG solution (2.5 mg/mL). The intraoperative application of ICG significantly enhanced the detection of bile leaks, leading to timely interventions and reduced postoperative complications.

Umemura et al. [163] described an unusual ICG fluorescence use in liver cyst surgery: they performed an Endoscopic Retrograde Cholangio-Pancreatography (ERCP) one day before surgery, placing a 5-Fr Endoscopic Nasal Biliary Drainage (ENBD) into the common hepatic duct. During transecting intrahepatic biliary branches, ICG (2.5 mg/mL) is injected into the biliary tract via ENBD. The ICG fluorescence imaging mode reveals intrahepatic biliary branches and unexpected small bile leakages. The authors concluded that ICG fluorescence clarifies that linear stapler sometimes fails the closure of biliary branches.

All included studies demonstrated the ability of ICG fluorescence to effectively identify bile leaks, contributing to improved surgical outcomes and reducing post-operative complications.

### 4.5. Liver Transplantation

ICG fluorescence imaging has been increasingly also utilized in liver transplantation to enhance visualization, assess graft quality, and predict postoperative outcomes. The following analysis discusses key studies that highlight the application and benefits of ICG fluorescence in liver transplantation.

Troisi et al. [157] presented the first case of a fully laparoscopic donor hepatectomy for pediatric liver transplantation using ICG fluorescence near-infrared fluorescence imaging in the Middle East. ICG fluorescence was injected intravenously (0.1 mg/kg) to visualize the left hepatic artery and biliary ducts within 10 s. Fluorescence imaging guided the precise cutting of the biliary ducts, enhancing visualization of biliary structures, improving the precision of the surgery, and reducing the risk of postoperative complications.

The study by Tomassini et al. [158] explores ICG fluorescence cholangiography as a method to improve visualization of intra-hepatic ducts during Pure Laparoscopic Living Donor Hepatectomy (PLDH). Overall, 11 consecutive donors underwent full laparoscopic left hepatectomy. Five different protocols of ICG-NIR-FC were tested to find the optimal dose, timing, and administration route for visualizing the biliary ducts (Table S4). The protocols varied in ICG fluorescence dosage, timing of administration relative to parenchymal transection, and whether the ICG fluorescence was administered intravenously or through the cystic duct. The effectiveness of each protocol was assessed by comparing standard preoperative and intraoperative cholangiography images with ICG-NIR-FC images. Despite two protocols (B and E) providing outcomes comparable to standard intraoperative cholangiography, ICG fluorescence did not show clear superiority in identifying segmental small ducts, particularly due to the limited penetration depth of NIR light (5–10 mm).

The Panaro et al. [159] study evaluates ICG fluorescence angiography during liver and pancreas transplantation to guide intraoperative decisions. The study focuses on ICG fluorescence use in assessing graft biliary duct perfusion in liver transplants and duodenal stump perfusion in pancreas transplants. Both in liver and in pancreatic transplantation, ICG fluorescence was injected intravenously at 0.5 mg/kg. In two out of six (33.33%) liver transplantations, the intraoperative strategy was changed based on ICG fluorescence findings, leading to additional graft bile duct resections, while one of five (20%) pancreas transplantations required duodenal stump resection due to ischemia detected by ICG fluorescence.

The procedure extended the operative time by approximately 10 min in liver transplantation and 8 min in pancreas transplantation. The authors concluded that ICG fluorescence angiography provides real-time objective information on organ perfusion, which is crucial for reducing ischemic complications in transplantation.

Kim et al. [160] investigated ICG near-infrared fluorescence imaging to enhance liver midplane demarcation during PLHD. This retrospective analysis compared 30 donors who underwent PLDH without ICG fluorescence to 46 donors with ICG fluorescence. The authors conclude that ICG fluorescence improves the accuracy of liver dissections and reduces operation times, thus enhancing overall surgical outcomes. Furthermore, a single low-dose injection of ICG fluorescence was sufficient for both liver midplane dissection and bile duct visualization, with no need for additional injections or intraoperative cholangiography.

### 4.6. Other Applications

Kawaguchi et al. [166] described the technical details of bile duct visualization during laparoscopic surgery. They administered 1 mL of intravenously ICG fluorescence. Following ICG fluorescence injection, a 0-degree laparoscope was introduced through a 12-mm trocar and placed above the hepatoduodenal ligament or hilar plate. The bile ducts were identified in several conditions: identification of the confluence of the left and right hepatic ducts before dissection, identification of the left hepatic duct with severe adhesions during dissection, and identification of the peripheral bile duct with severe adhesions during parenchymal transection. The authors compared ICG fluorescence to radiographic cholangiography and concluded that ICG fluorescence imaging may save time and avoid bile duct injury due to the insertion of a tube for the injection of contrast material.

In 2020, Tanaka et al. [167] reported a case of HCC treated with laparoscopic anatomical liver. Intraoperative liver segmentation is necessary to accomplish laparoscopic anatomical liver resection. One day before surgery, surgeons performed hepatic artery embolization with an ICG-Lipiodol^®^ mixture and Gelpart^®^ to define the region for hepatectomy. Gelpart^®^ prevents ICG from being quickly washed out due to blood flow. This technique allows better visualization of target segments interested in HCC. Nevertheless, Tanaka et al. reported some limitations: firstly, tumor necrosis could be reported after Lipiodol^®^ injection due to its well-known anticancer effect. In addition, in the case of significant arteriovenous shunting or arterial communication, this approach might become more difficult. Although the ICG-Lipiodol^®^ mixture and Gelpart^®^ increase surgical costs, the authors reported lower hospital costs and post-operative complication rates when compared to traditional hepatectomy.

ICG fluorescence represents a key point of lymphography for colorectal cancer and Upper GastroIntestinal Malignancies [3,80,170]. As reported for gastric and breast cancer, several experiences in the literature describe the ICG fluorescence superiority when compared to radioactive tracers and dedicated probes to detect lymph nodes involved in metastatic disease. Indeed, ICG fluorescence shows a higher sensitivity in lymphatic channel evaluation involved in first tumor drainage.

Ruzzenente et al. [168] proposed the LIver SEntinel LYmph-node (LISELY) study that involved 18 patients who underwent surgery for liver and biliary duct neoplasms. The primary lymphatic outflow and the sentinel lymph node were identified by intraoperative NIR imaging with ICG fluorescence, which was detected at predetermined brief intervals. The lymphatic outflow route was visible in 14 patients (77.8%) at a median of 3 min (IQR 3–10) following ICG fluorescence injection. In 12 patients (66.7%), the sentinel lymph node was confirmed by histology testing. The authors concluded that this technique can be conducted safely and practically.

### 4.7. Cost Analysis

It is crucial to evaluate the associated costs to understand ICG fluorescence’s economic feasibility and the benefits that its application provides.

Direct ICG fluorescence costs depend on the supplier and the healthcare facility’s purchasing agreements. Many experiences in the literature reported costs between USD 50 to USD 250 [171,172,173]. Although the primary cost is represented by the imaging Equipment, NIR systems are mandatory to visualize ICG fluorescence and its initial purchase prices range from USD 50,000 to USD 150,000 [171], depending on the model and capabilities. Maintenance and calibration of these systems also incur additional costs.

Nevertheless, ICG fluorescence might reduce the operative time, enhancing anatomical visualization and tumor resection. For instance, Marino et al. [137,174] reported that ICG fluorescence use during robotic liver resection led to a 100% R0 resection rate, with improved detection of lesions that were missed by traditional imaging techniques [137]. The enhanced accuracy and reduced need for repeat surgeries can lead to significant cost savings over time.

According to experiences in the literature [26,108,175], these findings are directly linked to better perioperative outcomes, such as lower intra-operative and post-operative complication rates and shorter hospital stays. This, in turn, reduces the costs associated with extended hospitalizations and additional treatments.

### 4.8. Limitations

Despite its numerous advantages, ICG fluorescence imaging has several limitations that warrant consideration. Firstly, ICG fluorescence depends on adequate liver function and biliary excretion pathways; thus, its efficacy may be compromised in patients with severe liver dysfunction or cholestasis.

Technical limitations such as tissue depth penetration and background fluorescence interference can also affect the accuracy of ICG fluorescence imaging, particularly in obese patients or those with significant intra-abdominal scarring.

Secondly, while ICG fluorescence provides valuable information on vascular perfusion, it does not substitute for detailed anatomical knowledge or tactile feedback during surgery. Moreover, the cost associated with ICG fluorescence imaging systems and the need for specialized training in interpretation and utilization may limit its widespread adoption in resource-constrained settings.

Another key point is represented by serious allergic reactions or anaphylaxis that could be investigated in further RCT.

## 5. Conclusions

This systematic review highlights the significant role of ICG fluorescence in liver surgery, emphasizing its multifaceted applications such as tumor identification, segmentation, bile leak evaluation, metastatic lymph node detection, and liver transplantation. These advantages contribute to reduced operative time and minimized complications compared to traditional invasive techniques. Despite its established safety and effectiveness, challenges remain in the variability of different tumor types or varying degrees of liver dysfunction, particularly in complex HPB surgery settings. Future studies should aim to address the current limitations and expand the evidence base for broader clinical application, ultimately enhancing patient outcomes in hepatic surgeries.

## Figures and Tables

**Table 1:** Patients, intervention, comparison, outcomes, and study design method used to screen experiences in the literature.

**Population**	Patients Who Underwent Open, Laparoscopic, or Robotic Liver Surgery
**Intervention**	Dose and timing of ICG administration for benign and malignant liver neoplasm or cysts
**Comparator**	Different protocols of administration in terms of dose and timing
**Outcomes**	Tumor detection, segmentation, bile leak, lymph nodes identifications, and vascular and biliary anatomy recognition
**Study design**	Randomized Clinical Trials (RCTs), controlled cohort studies, case series, and case reports which met inclusion criteria

## Data Availability

The raw data supporting the conclusions of this article will be made available by the authors, with undue reservation.

## Supplementary Materials

The following supporting information can be downloaded at https://www.mdpi.com/article/10.3390/cancers17050872/s1: Table S1. Protocols and applications of Indocyanine Green Fluorescence to identify primary and secondary liver tumors; Table S2. Protocols and applications of Indocyanine Green Fluorescence to liver segment identifications; Table S3. Protocols and applications of Indocyanine Green Fluorescence to liver segment identifications and tumor detection; Table S4. Protocols and applications of Indocyanine Green Fluorescence during liver transplant; Table S5. Protocols and applications of Indocyanine Green Fluorescence to bile leak identifications; Table S6. Protocols and applications of Indocyanine Green Fluorescence in particular applications.
