# Supplementary material for: Real-Time Navigation in Liver Surgery Through Indocyanine Green Fluorescence: An Updated Analysis of Worldwide Protocols and Applications
Source: Cancers (Basel). 2025 Mar 3;17(5):872. doi: 10.3390/cancers17050872 (PMC11898688; doi:10.3390/cancers17050872)
Supplement: Supplementary file 1 [file cancers-17-00872-s001.zip › cancers-3454390-supplementary.pdf]

cancers

MDPI

Systematic Review

Real-Time Navigation in Liver Surgery Through Indocyanine Green Fluorescence: An Updated Analysis of Worldwide Protocols and Applications

Pasquale Avella [1,2,3,*], Salvatore Spiezia [3], Marco Rotondo [3], Micaela Cappuccio [1], Andrea Scacchi [4], Giustiniano Inglese [3], Germano Guerra [3], Maria Chiara Brunese [3,*], Paolo Bianco [2], Giuseppe Amedeo Tedesco [3], Graziano Ceccarelli [5,6,†] and Aldo Rocca [2,3,†]

1 Department of Clinical Medicine and Surgery, University of Naples "Federico II", 80138 Naples, Italy
2 Hepatobiliary and Pancreatic Surgery Unit, Department of General Surgery, Pineta Grande Hospital, 81030 Castel Volturno, Italy
3 Department of Medicine and Health Science "V. Tiberio", University of Molise, 86100 Campobasso, Italy
4 School of Medicine and Surgery, University of Milano-Bicocca, 20126 Monza, Italy
5 Division of General and Minimally Invasive Surgery, Department of Surgery, San Giovanni Battista Hospital, 06034 Foligno, Italy
6 Minimally Invasive and Robotic Surgery Unit, San Matteo Hospital, 06049 Spoleto, Italy
* Correspondence: avella.p@libero.it (P.A.); mariachiarabrunese@gmail.com (M.C.B.); Tel.: +39-331-2360332 (P.A.)
† These authors contributed equally to this work.

Table S1. Protocols and applications of Indocyanine Green Fluorescence to identify primary and secondary liver tumours. Abbreviations: HCC, Hepatocellular Carcinoma; CCA, Cholangiocarcinoma; CRLM, Colorectal Liver Metastases; PLC, Primary Liver Cancer; FNH, Focal Nodular Hyperplasia; GIST, gastrointestinal stromal tumour, SCC, Squamous Cell Carcinoma; LT, Liver Transplant; ICG, Indocyanine Green Fluorescence; NIR, near-infrared camera; PDE, Photodynamic Eye (Hamamatsu Photonics Co., Shizuoka, Japan); PIN, PINPOINT (Stryker Co., Michigan, US); HEMS, HyperEye Medical System (Mizuho Co., Ltd., Tokyo, Japan); Visera, Visera Elite II (Olympus Co., Tokyo, Japan); Image 1/Image 1S, Image 1/Image1 S camera systems (KARL STORZ SE and Co. KG, Tuttlingen, Germany); STORZ, KARL STORZ SE and Co. KG (Tuttlingen, Germany); PS, Positive Staining; NS, Negative Staining; NA, Not Available.

Author	Year	Study type	Number of patients	Type of surgery (Open, Minimally Invasive Surgery)	Type of Laparoscopic System	Type of Robot System	Type of NIR	Disease (HCC, CCA, CRLM or others)	Age (mean, median ±SD or range)	Cirrhosis (%)	Dose of ICG (mg/kg, ml)	Timing of administration	Type of navigation (Tumor detection, Segmentation, both)	Other uses	Major hepatectomy (%)	SIGN
Gotoh *et al. [6]*	2009	Retrospective study	10	Open	\	\	PDE	HCC	67 (55-76)	50.0	0.5 mg/kg	Preoperative: 4.8 (1-8) days prior	Tumor detection	\	NA	LOW
Ishizawa *et al.[13]*	2009	Retrospective study	26	Open	\	\	PDE	HCC, CRLM	NA	NA	0.5 mg/kg	Preoperative: 1 to 7 days (median, 3 days) in patients with HCC and from 1 to 14 days (median, 3 days) in patients with metastasis	Tumor detection	\	NA	LOW
Uchiyama *et al.[39]*	2010	Retrospective study	32	Open	\	\	PDE	CRLM	71.8 ± 9.3	NA	0.5 mg/kg	Preoperative: 2 weeks prior	Tumor detection	\	NA	ACCEPT
Ishizuka *et al.[40]*	2012	Prospective study	7	Open	\	\	PDE	CRLM	64.7 ± 8.5	NA	0.1 mg/kg	Preoperative: 6.1 ± 5.1 days prior	Tumor detection	\	0	LOW
Peloso *et al.[41]*	2013	Retrospective study	25	Open	\	\	PDE	CRLM	61.5 (42–87)	NA	0.5 mg/kg	Preoperative: 24 h prior	Tumor detection	\	NA	ACCEPT
Satou *et al.[42]*	2013	Retrospective study	17	Open	\	\	PDE	HCC	66 (55–78)	NA	0.5 mg/kg	Preoperative: 3 (1–24) days prior	Tumor detection	\	NA	LOW
Morita *et al.[43]*	2013	Retrospective study	58	Open	\	\	PDE	HCC	71 ± 8.4	NA	0.5 mg/kg	Preoperative: 3–28 days prior	Tumor detection	\	NA	ACCEPT
van der Vorst *et al.*[44]	2013	Randomized Clinical Trial	40	Open	\	\	Others	CRLM	63 (45-77)	0	10 or 20 mg	Preoperative: 24 or 48 hours prior	Tumor detection	\	15	HIGH
Kudo *et al.[45]*	2014	Case series	17	Minimally Invasive Surgery	NA	\	Olympus	HCC, CRLM	NA	NA	0.5 mg/kg	Preoperative: within 2 weeks	Tumor detection	\	NA	LOW
Ishizawa *et al.[10]*	2014	NA	170	Open	\	\	PDE	HCC	NA	64.1	0.5 mg/kg	Preoperative: within 2 weeks	Tumor detection	\	NA	HIGH
Tanaka *et al.[46]*	2014	NA	33	Open	\	\	PDE	PLC, CRLM	61 and 67.5	27.2	0.5 mg/kg	Preoperative: > 2 days prior	Tumor detection	\	NA	ACCEPT

Tummers *et al.*[47]	2014	Randomized Clinical Trial	3	Minimally Invasive Surgery	NA	\	STORZ	Melanoma	68.6 (75, 66, 65)	NA	10 mg	Preoperative: 1 day prior	Tumor detection	\	0	LOW
Kawaguchi *et al.*[48]	2015	Case report	2	Minimally Invasive Surgery	NA	\	Olympus	HCC, CRLM	69.5 (66, 73)	50	0.5 mg/kg	Preoperative: 2 weeks prior	Tumor detection	\	0	LOW
Shimada *et al.*[49]	2015	Retrospective study	19	Open	\	\	PDE	HCC, CRLM	65	26	0.5 mg/kg	Preoperative: 2-14 days prior	Tumor detection	To evaluate the liver tissue; also performed intraoperative ICG injection in several cases	0	LOW
Takahashi *et al.*[50]	2016	Prospective study	15	Minimally Invasive Surgery	NA	NA	Firefly, PIN	CRLM, FNH	55.7 (37-76)	NA	5 or 7.5 mg	1-2 days prior or intraoperative IV	Tumor detection	\	0	LOW
Kaibori *et al.*[51]	2016	NA	48	Open	\	\	PDE	PLC, CRLM	72±9	NA	0.5 mg/kg	Preoperative: 14 days prior	Tumor detection	\	NA	LOW
Boogerd *et al.*[52]	2016	NA	22	Minimally Invasive Surgery	STORZ	\	STORZ	HCC, CRLM, others	65 (28-76)	14	10 mg	Preoperative: 24 h prior	Tumor detection	\	NA	LOW
Barabino *et al.*[53]	2016	NA	3	Open	\	\	Others	CRLM	NA	NA	0.25 mg/kg	Preoperative: 24 h prior	Tumor detection	\	NA	LOW
Kaibori *et al.*[54]	2016	Retrospective study	190	NA	\	\	Clairvivo OPT (Shimadzu, Kyoto, Japan	HCC	HC group: 68.8 ± 9.3 HS group: 69.6 ± 9.3	HC group: 58 (50 %) HS group: 1 (1 %)	0.5 mg/kg	Preoperative: 1–8 weeks prior to surgery	Tumor detection	\	\	ACCEPT
Handgraaf *et al.*[55]	2017	Retrospective study	67	Open	\	\	Others	CRLM	62 ± 9.2	NA	10 or 20 mg	Preoperative: 1 or 2 days prior	Tumor detection	\	10	ACCEPT
Masuda *et al.*[56]	2017	Prospective study	33	Open	\	\	Others	HCC	53 ± 9.2	100	0.05 mg/kg	Preoperative: 1 week prior	Tumor detection	\	\	ACCEPT
Benedicenti *et al.*[57]	2018	Case report	1	Open	\	\	PDE	Metastases of SCC	74	NA	0.5 mg/kg	Preoperative: 10 days prior	Tumor detection	\	0	LOW
Nanashima *et al.*[31]	2018	Case report	3	Open	\	\	PDE	HCC	75.3 (66, 84, and 76)	33.3	0.5 mg/kg	Preoperative: several days prior	Tumor detection	\	67	LOW

Lieto *et al.*[58]	2018	Retrospective study	9	Open	\	\	Others	HCC, CRLM	65 (64-79)	NA	0.5 mg/kg	Preoperative: 24 h prior	Tumor detection	\	NA	LOW
Aoki *et al.*[59]	2018	Comparative study	25	Minimally Invasive Surgery	NA	\	PIN	HCC, CRLM	63 (34–84)	0	0.5 mg/kg of ICG (Diagnogreen; Daiichi Sankyo, Tokyo, Japan)	Preoperative: 2-14 days before surgery	Tumor detection	\	\	ACCEPT
Yoshioka *et al.*[60]	2019	Case report	1	Minimally Invasive Surgery	NA	\	NA	HCC	73	100	0.5 mg/kg	Preoperative: 2 days prior	Tumor detection	\	0	LOW
Alfano *et al.*[61]	2019	Retrospective study	27	Open	\	\	IMAGE 1S	PLC, CRLM	68.2 ± 9.0	48.2	0.5 mg/kg	Preoperative: within 7 days (>7 days, additional IV)	Tumor detection	\	15	LOW
Souzaki *et al.*[62]	2019	Retrospective study	10 lesions	Open	\	\	D-LIGHT P	PLC and LT	NA	NA	0.5 mg/kg	Preoperative: 0.5 ± 33.7 h	Tumor detection	\	60	ACCEPT
He *et al.*[63]	2019	Case report	2	Open	\	\	Key Laboratory of Molecular Imaging in Beijing	HCC	56.5	100	0.5 mg/kg	Preoperative: 72-96 h before surgery	Tumor detection	\	NA	ACCEPT
Shirakawa *et al.*[64]	2019	Prospective study	40	Minimally Invasive Surgery	NA	\	(Karl Storz GmbH & Co. KG	Liver metastases of pancreatic cancer	NA	NA	0.5 mg/kg	Preoperative: 1 day before surgery	Tumor detection	\	\	LOW
Li *et al.*[65]	2020	Retrospective study	23	Minimally Invasive Surgery	\	Da Vinci Si	NA	FNH	30.5	NA	0.25 mg/kg	Preoperative: 48 h prior	Tumor detection	\	0	LOW
Achterberg *et al.*[66]	2020	Retrospective study	16 lesions	Minimally Invasive Surgery	Olympus, Striker	Da Vinci	Visera, PIN, Firefly	CRLM	NA	NA	10 mg	Preoperative: 24 h prior	Tumor detection	\	0	LOW
Li *et al.*[67]	2020	Case report	1	Minimally Invasive Surgery	NA	\	NA	HCC	57	NA	0.25 mg/kg	Preoperative: 72 h prior	Tumor detection	\	0	LOW
Tashiro *et al.*[68]	2020	Retrospective study	52	Open	\	\	PIN	HCC, CRLM	72 and 67.5 (34-85)	3.8	0.5 mg/kg	Preoperative: 2-14 days prior	Tumor detection	\	NA	ACCEPT
He *et al.*[69]	2020	Randomized Clinical Trial	21	Minimally Invasive Surgery	NA	\	Others	Hepatolithiasis	53.8 ± 15.0	NA	0.25 mg/kg	Intraoperative: not detailed	Tumor detection	\	0	ACCEPT
Mehdorn *et al.*[70]	2021	Retrospective study	20	Minimally	\	Da Vinci Xi	Firefly	HCC, CRLM	64.0 ± 12.3	NA	0.32 ± 0.08 mg/kg	Preoperative:	Tumor detection	\	5	ACCEPT

				Invasive Surgery								24 h prior				
Matsumura *et al.*[71]	2021	Case report	1	Open	\	\	PIN	HCC	72	NA	2.5 mg	Preoperative: 1 day prior	Tumor detection	\	100	LOW
Bijlstra *et al.*[72]	2021	Case report	2	Open	\	\	Others	GIST	75.5 (69 and 82)	NA	10 mg	Preoperative: 24 h prior	Tumor detection	\	0	LOW
Kobayashi *et al.*[73]	2021	Retrospective study	154 lesions in 82 patients	Open	\	\	PDE-NEO, PIN	HCC, others	0.25 mg-IV: 58 (40–73) 1.25 mg-IV: 69 (32–82) 2.5 mg-IV: 73 (37–87) 3.75 mg-IV: 69 (46–79)	46.3 (Cirrhosis, Chronic Epatitis)	0.5 mg/kg: for ICG retention test 0.25 mg, 1.25 mg, 2.5 mg, 3.75 mg	Preoperative	Tumor detection	\	\	HIGH
Cho *et al.*[74]	2021	Retrospective study	17	Open, Minimally Invasive Surgery	NA	\	D-Light P, Karl Storz SE	Hepatoblastoma	18 months (range, 4–140 months)	NA	0.3 mg/kg	Preoperative: 24-48 h before the surgery	Tumor detection	\	\	ACCEPT
Han *et al.*[75]	2021	Retrospective study	4	Minimally Invasive Surgery	NA	\	PIN	CCA	69	NA	5 mg	Preoperative: 24 h before the surgery	Tumor detection	\	\	LOW
Whitlock *et al.*[76]	2021	Retrospective study	14	Open, Minimally Invasive Surgery	NA	\	STORZ Image1™, MEDTRONIC Elevision™, and the STRYKER SPY™	HCC, Hepatoblastoma, others	NA	NA	0.2-0.75 mg/kg	Preoperative: 24–96 h before surgery	Tumor detection	\	\	ACCEPT
Lake *et al.*[77]	2021	Retrospective study	29	Open, Minimally Invasive Surgery	NA	\	Hamamatsu PDE-NEO, Stryker SPY-PHI, 1588-AIM system	Hepatoblastoma	3.64	NA	0.5 mg/kg	Preoperative: 1–6 days prior to surgery (liver resection) 24 h prior to surgery (metastatectomy)	Tumor detection	\	\	ACCEPT
Gerber *et al.*[78]	2022	Retrospective study	17	Minimally Invasive Surgery	NA	\	PIN	HCC	63 ± 4.49	NA	Intraoperative: 0.3125 mg + Second	Intraoperative	Tumor detection	\	NA	ACCEPT

											dose of 0.3125 mg if needed					
Tashiro *et al.*[12]	2022	Retrospective study	3	Open	\	\	BZ-X800 fluorescence microscope (Keyence, Osaka, Japan) using an OP-87767 ICG filter (Keyence)	HCC, CRLM, Lung carcinoid liver metastases	72.5 (65-83)	NA	0.5 mg/kg	Preoperative: 2-14 days before the surgery	Tumor detection	\	\	LOW
Seo *et al.*[79]	2022	Case report	1	Minimally Invasive Surgery	NA	\	NA	CRLM	75	NA	0.5 mg/kg	Preoperative: 48 h before the surgery	Tumor detection	\	\	LOW
Feng *et al.*[80]	2022	Retrospective study	11	Open, Minimally Invasive Surgery	NA	Da Vinci	OPTO-CAM2100 (Guangdong Optomedic Technologies, Inc.), FLI-10B (Nanjing Nuoyuan Medical Devices Co., Ltd.), DPM-III-01 (Zhuhai Dipu Medical Technology Co., Ltd.) and the Da Vinci Surgical System	Hepatoblastoma, Calcified nested stromal-epithelial tumor, Yolk sac tumor, undifferentiated embryonal sarcoma of liver	48 months	NA	from 0.1 to 0.2 mg/kg	Preoperative: 24 h before the surgery	Tumor detection	\	\	ACCEPT
Shen *et al.*[81]	2022	Retrospective study	16	Open	\	\	NA	Hepatoblastoma	15 months (8–134 months)	NA	0.5 mg/kg	Preoperative: 48-72 h before surgery	Tumor detection	\	NA	LOW

Hu *et al.*[82]	2022	Retrospective study	29	Minimally Invasive Surgery	Guangdong Optomedic Technologies, Inc.	\	NA	HCC	58.17±9.99	NA	0.3-0.5 mg/kg	Preoperative: 2-3 days before surgery	Tumor detection	\	\	LOW
Wang *et al.*[83]	2022	Retrospective study	14	Minimally invasive Surgery	NA	\	NA	NETs liver metastases	54.5 (46–60)	NA	0.1–0.5 mg/kg	Preoperative: 1 to 3 days before surgery	Tumor detection	\	0	ACCEPT
Itoh *et al.*[84]	2022	Retrospective study	55 (32 using propensity score matching)	Minimally Invasive Surgery	NA	\	The Visera Elite II (Olympus, Tokyo, Japan) or IMAGE 1 S Camera Systems (KARL STORZ, El Segundo, CA, USA)	HCC, CCA, metastatic liver tumor	67 (44–83)	NA	5 mg/kg	Preoperative: 2–7 days prior to surgery	Tumor detection	\	\	ACCEPT
Peng *et al.*[85]	2022	Case report	1	Minimally Invasive Surgery	NA	\	NA	Liver metastases of rectal neuroendocrine tumor	58	0	0.5 mg/kg	Preoperative: 24 h before surgery	Tumor detection	\	\	LOW
Rompianesi *et al.*[86]	2023	Prospective study	Preoperatively in 61 patients	Minimally Invasive Surgery	\	Da Vinci Xi	Firefly	HCC, CRLM, CCA, others	64.8 (20–84)	NA	Preoperative: 0.25–0.50 mg/kg	Preoperative: 2-4 days before the surgery	Tumor detection	\	\	ACCEPT
Shen *et al.*[87]	2023	Retrospective study	23	Open	\	\	NIF imaging system (Real-IGS, Nuoyuan Medical Equipment Co., Ltd., Nanjing, China	Hepatoblastoma	31 months	NA	0.1 mg/kg	Preoperative: 24-48 h before surgery	Tumor detection	\	43.47	LOW
Sebagh *et al.*[88]	2023	Retrospective study	16	Open	\	\	The Fluobeam® (Fluoptics SAS, Grenobl	HCC	68 (48–81)	NA	0.2 mg/kg body weight	Preoperative: about 18 h before surgery	Tumor detection	\	\	ACCEPT

							e, France)								
Liu *et al.[89]*	2023	Retrospective study	22	Open, Minimally Invasive Surgery	FLI-10B (Nanjing Nuoyuan Medical Devices Co., Ltd.), OPTO–CAM2100 (Guangdong Optomedic Technologies, Inc.), DPMIII-01 (Zhuhai Dipu Medical Technology Co., Ltd.)	Da Vinci Surgical System	FLI-10B (Nanjing Nuoyuan Medical Devices Co., Ltd.), OPTO–CAM2100 (Guangdong Optomedic Technologies, Inc.), DPMIII-01 (Zhuhai Dipu Medical Technology Co., Ltd.)	Hepatoblastoma	32.5 (15-45.3)	NA	0.1–0.5 mg/kg	Preoperative 1-3 days before surgery	Tumor detection	\	NA

ACCEPT

Table S2. Protocols and applications of Indocyanine Green Fluorescence to liver segment identifications. Abbreviations: HCC, Hepatocellular Carcinoma; CCA, Cholangiocarcinoma; CRLM, Colorectal Liver Metastases; PLC, Primary Liver Cancer; FNH, Focal Nodular Hyperplasia; GIST, gastrointestinal stromal tumour, SCC, Squamous Cell Carcinoma; LT, Liver Transplant; ICG, Indocyanine Green Fluorescence; NIR, near-infrared camera; PDE, Photodynamic Eye (Hamamatsu Photonics Co., Shizuoka, Japan); PIN, PINPOINT (Stryker Co., Michigan, US); HEMS, HyperEye Medical System (Mizuho Co., Ltd., Tokyo, Japan); Visera, Visera Elite II (Olympus Co., Tokyo, Japan); Image 1/Image 1S, Image 1/Image1 S camera systems (KARL STORZ SE and Co. KG, Tuttlingen, Germany); STORZ, KARL STORZ SE and Co. KG (Tuttlingen, Germany); PS, Positive Staining; NS, Negative Staining; NA, Not Available.

Author	Year	Study type	Number of patients	Type of surgery (Open, Minimally Invasive Surgery)	Type of Laparoscopic System	Type of Robot System	Type of NIR	Disease (HCC, CCA, CRLM or others)	Age (mean, median ±SD or range)	Cirrhosis (%)	Dose of ICG (mg/kg, ml)	Timing of administration	Type of navigation (Tumor detection, Segmentation, both)	Other uses	Major hepatectomy (%)	SIGN
Aoki *et al.*[1]	2008	Retrospective study	35	Open	\	\	PDE-2	HCC, CCA, CRLM, others	66 (46–80)	34.28	One milliliter of ICG (Wako Pure Chemical Industries, Osaka, Japan) (5 mg/ml)	Intraoperative: NA	Segmentation	\	\	HIGH
Aoki *et al.*[90]	2009	Retrospective study	81	Minimally Invasive Surgery	NA	\	PDE	PLC, Metastases	69 (46-87)	18.5	5 mg	Intraoperative: PS	Segmentation	\	NA	ACCEPT
Uchiyama *et al.*[91]	2011	Retrospective study	22	Open	\	\	PDE	HCC	73.5 ± 9.8	100	0.5 mg/kg	Intraoperative: NS	Segmentation	\	36	ACCEPT
Ishizawa *et al.*[92]	2012	NA	2	Minimally Invasive Surgery	NA	\	NA	NA	NA	NA	0.025 mg for PS 2.5 mg for NS	Intraoperative: PS and NS	Segmentation	\	NA	LOW
Sakoda *et al.*[93]	2014	Case report	2	Minimally Invasive Surgery	NA	\	Olympus	HCC	75 (72, 78)	100	5 mg	Intraoperative: PS	Segmentation	\	0	LOW
Kurihara *et al.*[94]	2015	Case report	2	Open	\	\	HEMS	CCA, CRLM	68 (78, 58)	0	0.25 mg/kg	Intraoperative: NS	Segmentation	\	100	LOW

Inoue *et al.*[95]	2015	Case report	24	Open	\	\	HEMS	PLC, CRLM	70(51-86)	16	2.5 mg	Intraoperative: PS and NS	Segmentation	\	25	HIGH
Miyata *et al.*[96]	2015	NA	30	Open	\	\	PDE	PLC, CRLM	NA	20	0.25 mg	Intraoperative: PS	Segmentation	\	7	ACCEPT
Inoue *et al.*[97]	2015	Prospective study	24	Open	\	\	HyperEye Medical System, Mizuho Ika-kogyo Co	HCC, CRLM, CCA	70 (51–86)	16.8	2.5 mg	Preoperative ICG-R15: 28 h (27–170) before surgery and intraoperative: NA	Segmentation	\	0	HIGH
Kobayashi *et al.*[98]	2017	NA	105	Open	\	\	PDE	PLC, Metastases	68 (17-83)	14.3	0.25 mg	Intraoperative: PS and NS	Segmentation	\	10	HIGH
Kawaguchi *et al.*[99]	2017	NA	21	Open	\	\	PDE	HCC, CRLM	67 (38–84)	19.0	2·5 μg per ml Total Liver Volume)	Intraoperative: NS	Segmentation	\	33	LOW
Meng *et al.*[100]	2018	Case report	1	Minimally Invasive Surgery	NA	\	PIN	Healthy liver in LT	34	0	1.0 mg	Intraoperative: PS	Segmentation	\	100	LOW
Nomi *et al.*[101]	2018	Retrospective study	16	Minimally Invasive Surgery	NA	\	PIN	NA	NA	NA	1.5 mg	Intraoperative: NS	Segmentation	\	13	LOW
Nishino *et al.*[102]	2018	Retrospective study	23	Open	\	\	Others	PLC, CRLM	66.9 ± 10.7	NA	0.25 mg	Intraoperative: PS and NS	Segmentation	\	61	LOW
Yang *et al.*[103]	2018	Prospective study	27	Open	\	\	PINPOINT™ imaging system (NOVADAQ, Toronto, Canada)	HCC, CCA, Metastatic disease, focal nodular hyperplasia	48.9 ± 12.6	29.6	Intravenous (IV): 2.5 mg PV: 0.25 mg	Intraoperative: NA	Segmentation	\	\	ACCEPT
Ueno *et al.*[104]	2018	Retrospective study	10	Minimally Invasive Surgery	NA	\	PIN	HCC, CRLM	74 ± 7	NA	0.25 mg	Intraoperative: PS	Segmentation	\	0	LOW
Chiba *et al.*[105]	2019	Retrospective study	24	Open	\	\	HEMS	Gallbladder Ca	73 (56-82)	NA	5 mg	Intraoperative: PS	Segmentation	\	0	LOW
Ueno *et al.*[106]	2019	Retrospective study	10	Minimally Invasive	NA	\	PIN	HCC, CRLM	73.6	NA	2 mL diluted ICG saline solution	Intraoperative: NA	Segmentation	\	\	LOW

				Surgery							(0.125 mg/mL, Daiichi Sankyo, Japan)					
Nanashima *et al.*[107]	2019	Prospective study	40	Open	\	\	PDE Hamamatsu Photonics	HCC, CCA, CRLM, benign lesions	68.6 ± 9.1	17.5	0.25 mg/mL	Intraoperative: PS	Segmentation	\	2.08	ACCEPT
Xu *et al.*[108]	2020	Retrospective study	36	Minimally Invasive Surgery	NA	\	PIN	PLC, others	58 (34-74)	67	1.25-2.5 mg for PS 2.5 mg for NS	Intraoperative: PS and NS	Segmentation	\	42	LOW
Kubo *et al.*[109]	2020	Retrospective study	12	Open	\	\	HEMS, PIN	PLC, CRLM	67.3 ± 9.0	NA	2.5 mg	Intraoperative: NS	Segmentation	\	100	LOW
Ito *et al.*[110]	2020	Retrospective study	3	Minimally Invasive Surgery	NA	\	PIN	HCC, CRLM	71.7 ± 6.0	NA	0.25 mg	Intraoperative: PS	Segmentation	\	0	LOW
Pansa *et al.*[111]	2020	Case report	1	Open	\	\	PIN	HCC	71	NA	7.5 mg	Intraoperative: NS	Segmentation	\	0	LOW
Urade *et al.*[112]	2020	Retrospective study	3	Minimally Invasive Surgery	NA	\	Visera, PIN	HCC, CRLM	64 ± 18	33.3	2.5 mg	Intraoperative: NS	Segmentation	\	0	LOW
Aoki *et al.*[113]	2020	Retrospective study	14	Minimally Invasive Surgery	NA	\	PIN	HCC, CRLM	64(42-85)	NA	0.025 mg	Preoperative: PS	Segmentation	\	0	LOW
Zhai *et al.*[114]	2020	Retrospective study	11	Minimally Invasive Surgery	NA	\	PIN	CCA	58.5	NA	1 mg	Intraoperative: NS	Segmentation	\	\	ACCEPT
Berardi *et al.*[115]	2021	Retrospective study	86	Minimally Invasive Surgery	NA	\	Visera, PIN	HCC, CRLM	69 (66-77)	59.3	0.5 mg	Intraoperative: NS	Segmentation	\	0	HIGH
Procopio *et al.*[116]	2021	Prospective study	15	Open	\	\	HEMS	HCC	71 (52-85)	86.7	0.5 mg/kg	Intraoperative: NS	Segmentation	\	0	LOW
Funamizu *et al.*[117]	2021	Retrospective study	74	Minimally Invasive Surgery	NA	\	PIN, VISERA, HOPKINS II	HCC, CRLM, CCA, others	69.3 ± 2.1	46	0.5 mg/body	Intraoperative: NS	Segmentation	\	0	ACCEPT
Yasuda *et al.*[118]	2021	Case report	1	Minimally Invasive e	NA	\	VISERA	HCC	69	NA	2.5 mg	Intraoperative: NS	Segmentation	\	\	LOW

				Surgery												
Li *et al.[119]*	2021	Retrospective study	8	Minimally Invasive Surgery	NA	\	NA	HCC	NA	NA	5 mL bolus of ICG with a concentration of 125 mg/mL	Intraoperative: PS	Segmentation	\	\	ACCEPT
Liang *et al.[120]*	2021	Retrospective study	43	Minimally Invasive Surgery	L-PMOD, UHS	\	PINPOINT	HCC, CCA, CRLM, others	55.4 ± 12.7	27.9	1 ml, 5 mg/L	Intraoperative: NS	Segmentation	\	0	ACCEPT
Ceccarelli *et al.[121]*	2022	Case report	1	Minimally Invasive Surgery	\	Da Vinci Xi	NA	CCA	77	0	0.5 mg	Intraoperative: PS and NS	Segmentation	Biliary tree visualization	0	ACCEPT
Kasai *et al.[122]*	2022	Case report	1	Minimally Invasive Surgery	NA	\	PIN	HCC	60	100	Intraoperative: 0.25 mg percutaneous tumor-bearing portal vein puncture for P8	Intraoperative: NA	Segmentation	\	NA	ACCEPT
Chen *et al.[123]*	2022	Case report	1	Minimally Invasive Surgery	NA	\	NA	HCC	65	100	0.5 mg/kg	Preoperative: 7 days prior NS	Segmentation	Hepatic bile duct visualization	\	LOW
Lan *et al.[124]*	2022	Retrospective study	24	Minimally Invasive Surgery	NA	\	PIN	HCC, Metastasis, Hepatolithiasis, Hemangioma, FNH and hyperplastic disease	60.04±9.94	NA	5 ml of 0.025 mg/ml ICG	Intraoperative NA	Segmentation	\	41.66	ACCEPT
Zhang *et al.*[125]	2022	Retrospective study	20	Minimally Invasive Surgery	NA	\	NOVADAQ	CCA	ICG Group (N=12) = 59 (53.3-64.5) ICG failed group (N=8) = 62 (50.5e68.0)	NA	0.025 mg/mL	Intraoperative NA	Segmentation	\	\	ACCEPT

Cheng *et al.[126]*	2022	Retrospective study	24	Minimally Invasive Surgery, conversion to open	NA	\	NA	HCC, CCA	55.5±12.5	33.3	2.5 mg	Intraoperative: NS	Segmentation	\	0	ACCEPT
Zhou *et al.[127]*	2023	Retrospective study	50	Minimally Invasive Surgery	NA	\	NA	HCC	58.26±10.30	66	1–5 ml	Intraoperative; PS and NS	Segmentation	\	NA	ACCEPT
Sun *et al.[128]*	2023	Case report	1	Minimally Invasive Surgery	NA	\	NA	HCC	53	NA	Percutaneous injection of 5 mL, 0.025 mg/mL of ICG into the S8 portal branch by using an 18G PTCD	Intraoperative: PS	Segmentation	\	\	LOW
Luján *et al.[129]*	2022	Case report	1	Minimally Invasive Surgery	NA	\	NA	Gallbladder Ca	73	NA	1.5 mg	Preoperative: NA	Segmentation	Hepatic bile duct visualization	\	LOW
Wan *et al.[130]*	2023	Case report	1	Minimally Invasive Surgery	NA	\	NA	HCC	49	100	1 ml, 5 mg/l	Intraoperative: NS	Segmentation	\	\	ACCEPT
Rompianesi *et al.[86]*	2023	Prospective study	Intraoperatively in 42 patients	Minimally Invasive Surgery	\	Da Vinci Xi	Firefly	HCC, CRLM, CCA, others	64.8 (20–84)	NA	Intraoperative: 2.5 mg in 2 mL of injectable solution in negative staining; 1.25 mg in 5 mL of injectable solution in positive staining	Intraoperative: PS and NS	Segmentation	\	\	ACCEPT
Jiang *et al.[131]*	2023	Retrospective study	21	Minimally Invasiv	NA	\	HyPixel™ R1, Mindray,	HCC, CRLM	60.7 ± 14.9	47.6	5-10 ml (depending on	Intraoperative: PS	Segmentation	\	\	

				e Surgery			Shenzhen, China)				the segmental volume) of 0.025 mg/ml					ACCEPT
Lin *et al.[132]*	2024	Retrospective study	12	Minimally Invasive Surgery	NA	\	PIN, DPM	NA	52.92 ± 14.94	NA	0.5 mg	Intraoperative: NS	Segmentation	\	\	ACCEPT

Table S3. Protocols and applications of Indocyanine Green Fluorescence to liver segment identifications and tumor detection. Abbreviations: HCC, Hepatocellular Carcinoma; CCA, Cholangiocarcinoma; CRLM, Colorectal Liver Metastases; PLC, Primary Liver Cancer; FNH, Focal Nodular Hyperplasia; GIST, gastrointestinal stromal tumour, SCC, Squamous Cell Carcinoma; LT, Liver Transplant; ICG, Indocyanine Green Fluorescence; NIR, near-infrared camera; PDE, Photodynamic Eye (Hamamatsu Photonics Co., Shizuoka, Japan); PIN, PINPOINT (Stryker Co., Michigan, US); HEMS, HyperEye Medical System (Mizuho Co., Ltd., Tokyo, Japan); Visera, Visera Elite II (Olympus Co., Tokyo, Japan); Image 1/Image 1S, Image 1/Image1 S camera systems (KARL STORZ SE and Co. KG, Tuttlingen, Germany); STORZ, KARL STORZ SE and Co. KG (Tuttlingen, Germany); PS, Positive Staining; NS, Negative Staining; NA, Not Available.

**Author**	**Year**	**Study type**	**Number of patients**	**Type of surgery (Open, Minimally Invasive Surgery)**	**Type of Laparoscopic System**	**Type of Robot System**	**Type of NIR**	**Disease (HCC, CCA, CRLM or others)**	**Age (mean, median ±SD or range)**	**Cirrhosis (%)**	**Dose of ICG (mg/kg, ml)**	**Timing of administration**	**Type of navigation (Tumor detection, Segmentation, both)**	**Other uses**	**Major hepatectomy (%)**	**SIGN**
Abo *et al.*[28]	2015	Comparative study	117	Open	\	\	PDE	PLC, CRLM	68.6 ± 11.6	12.8	Preoperative: 0.5 mg/kg Intraoperative: 1.25 mg	Preoperative: 4-7 days prior (>14 days, additional IV) Intraoperative: PS	Tumor detection + Segmentation	\	27	HIGH
Terasawa *et al.*[133]	2017	Retrospective study	41	Minimally Invasive Surgery	NA	\	PIN	HCC, CRLM	65 (35–93)	NA	Preoperative: 0.5 mg/kg Intraoperative: 1.25 mg	Preoperative: within 3 days Intraoperative: NS	Tumor detection + Segmentation	\	0	ACCEPT
Zhang *et al.*[134]	2017	Retrospective study	50	Minimally Invasive Surgery	NA	\	PDE	PLC, CRLM	54.18	4	0.25 mg/kg	Intraoperative: PS and NS	Tumor detection + Segmentation	\	NA	ACCEPT
Peyrat *et al.*[27]	2018	Prospective study	43	Open	\	\	Others	PLC, CRLM	66.2 (38–84)	NA	Preoperative:0.25 mg/kg Intraoperative: 0.016-1.25 mg	Preoperative: 1 day prior Intraoperative: PS	Tumor detection + Segmentation	\	NA	LOW
Cheung *et al.*[135]	2018	Retrospective study	20	Minimally Invasive Surgery	NA	\	PIN	HCC	60.5 (47–73)	100	Preoperative: 0.5 mg/kg Intraoperative: 0.025 mg	Preoperative: 10 days prior (>14 days, additional IV) Intraoperative: NS	Tumor detection + Segmentation	\	25	ACCEPT

Gon *et al.*[136]	2019	Prospective study	110	Open	\	\	PIN	NA	NA	NA	0.5 mg/kg	Preoperative: 2 days prior Intraoperative: NS	Tumor detection + Segmentation	\	NA	LOW
Marino *et al.*[137]	2019	Case-Matched study	25	Minimally Invasive Surgery	\	Da Vinci Si	CCD, PDE	HCC, CRLM, CCA	66.2 (35–78)	12	Preoperative: NA Intraoperative: 2.5 mg	Preoperative; Intraoperative; PS and NS	Tumor detection + Segmentation	\	44	ACCEPT
Zhou *et al.*[138]	2019	Retrospective study	21	Minimally Invasive Surgery	NA	\	PIN	HCC	5 ≥ 60 years 16 < 60 years	42.9	0.25 mg/kg	Preoperative: 3–5 days before surgery	Tumor detection + Segmentation	\	0	ACCEPT
Lu *et al.*[139]	2020	Retrospective study	57	Minimally Invasive Surgery	NA	\	PIN	PLC, Metastases	57.3 ± 12.2	24.6	0.5 mg/kg	Preoperative: 0-7 days prior Intraoperative: NS	Tumor detection + Segmentation	\	NA	HIGH
Li *et al.*[140]	2020	Retrospective study	9	Minimally Invasive Surgery	NA	\	PIN	Echinococcosis	36.4 ± 7.6	NA	Preoperative: 0.5 mg/kg Intraoperative: 2.5 mg	Preoperative: 3 days prior Intraoperative: NS	Tumor detection + Segmentation	\	0	LOW
Miyashita *et al.*[141]	2020	Retrospective study	15	Open	\	\	Others	PLC, CRLM	73 (52-82)	NA	Preoperative: 0.5 mg/kg Intraoperative: 0.025 mg for PS, 0.25mg for NS	Preoperative: 3-4 days prior Intraoperative: PS and NS	Tumor detection + Segmentation	\	0	LOW
Yao *et al.*[142]	2020	Retrospective study	18	Open	\	\	PDE	HCC	52.9 ± 12.1	NA	2.5 mg	Intraoperative: PS and NS	Tumor detection + Segmentation	\	100	HIGH
Zhang *et al.*[143]	2020	Randomized Clinical Trial	30	Minimally Invasive Surgery	NA	\	Others	PLC, FNH	55.7 ± 11.2	33.3	Preoperative: 0.05-0.10 mg/kg Intraoperative: 2.5mg	Preoperative: 24-72 h prior Intraoperative: PS and NS	Tumor detection + Segmentation	\	40	ACCEPT
Marino *et al.*[144]	2020	Retrospective study	40	Minimally Invasive Surgery	\	Da Vinci Xi	Firefly	PLC, CRLM	69.4 (38–79)	15	Preoperative: 0.5mg/kg Intraoperative: 0.25 mg for PS, 2.5 mg for NS	Preoperative: 5 days prior Intraoperative: PS and NS	Tumor detection + Segmentation	\	45	ACCEPT
Kose *et al.*[145]	2020	Prospective study	41	Open, Minimally Invasive	NA	NA	PIN (laparoscopic and open cases) Firefly	HCC, CRLM, others	58.8 ± 13.5	NA	Preoperative and intraoperative: three ml of the 2.5 mg/dL	Preoperative and intraoperative: 1-2 day prior Preopera	Tumor detection + Segmentation	\	0	ACCEPT

				Surgery			(robotic cases)				Preoperative: 7.5 mg	tive: 1 day prior: NA				
Zheng *et al.[146]*	2021	Case report	1	Minimally Invasive Surgery	NA	\	PIN	HCC	42	100	Preoperative: 0.5 mg/kg Intraoperative: 5 mg	Preoperative: 5 days prior Intraoperative: NS	Tumor detection + Segmentation	\	0	LOW
Li *et al.[147]*	2021	Retrospective study	26	Minimally Invasive Surgery	NA	\	PIN	HCC, CRLM	54.2	38.5	Preoperative: 0.5 mg/kg (non-anatomical hepatectomy) 0.025–0.050 mg/kg during the operation (anatomical hepatectomy)	Preoperative: 2–5 days before: surgery (non-anatomical hepatectomy): PS and NS	Tumor detection + Segmentation	\	\	LOW
Franz *et al.[26]*	2021	Retrospective study	18	Minimally Invasive Surgery	Karl Storz, (Tuttlingen, Germany)	Da Vinci Xi	Karl Storz, (Tuttlingen, Germany)	HCC, CCA, peritoneal HCC metastases, adenoma, CRLM	77 (47-86)	Liver cirrhosis/-fibrosis:	0.5 mg/kg	Preoperative: 2-10 days prior; PS	Tumor detection + Segmentation	Fluorescence angiography	8	LOW
Qiu *et al.[148]*	2022	Retrospective study	7	Minimally Invasive Surgery	NA	\	Optomedic	Hepatoblastoma	14 months (range, 13 days–36 months)	NA	0.5 mg/kg	Preoperative: 48h before the surgery: NA	Tumor detection + Segmentation	\	\	LOW
Liu *et al.[149]*	2023	Retrospective study	50	Minimally Invasive Surgery	NA	\	PIN	HCC	56.82 ± 10.41	56	Preoperative: 0.5 mg/kg Intraoperative: 0.125–0.25 mg for PS, 0.25–0.5 mg for NS	Preoperative: 2-4 days, 4-6 for cirrhosis; Intraoperative; PS and NS	Tumor detection + Segmentation	\	14	ACCEPT
Karmarkar *et al.[150]*	2023	Retrospective study	15	Open, Minimally Invasive Surgery	NA	\	SPY Portable Handheld Imager (SPY-PHI) for open surgery and PIN	HCC, CRLM	71 (36-86)	NA	0.02 to 0.05 mg/kg	Preoperative: 2-3 h before the surgery: NA	Tumor detection + Segmentation	\	6.67	LOW
Kasai *et al.[151]*	2023	Retrospective study	18	Minimally Invasiv	NA	\	PIN, IMAGE1 S™	HCC,CRLM,CCA	51-85	NA	0.028-0.083 mg per 100	Intraoperative: PS	Tumor detection +	\	22	LOW

				e Surgery							mL of liver volume		Segmentation			
Rompianesi *et al.*[86]	2023	Prospective study	Pre and intra-operatively in 25 patients	Minimally Invasive Surgery	\	Da Vinci Xi	Firefly	HCC, CRLM,CCA, others	64.8 (20–84)	NA	Preoperative: 0.25–0.50 mg/kg Intraoperative: 2.5 mg in 2 mL of injectable solution in negative staining; 1.25 mg in 5 mL of injectable solution in positive staining	Preoperative: 2-4 days before the surgery and Intraoperative: PS and NS	Tumor detection + Segmentation	\	\	ACCEPT
Hardy *et al.*[152]	2023	Prospective study	24	Open, Minimally Invasive Surgery	NA	NA	PIN, Firefly, Elevision	CRLM, Benign liver cyst	61 ± 11.77	NA	0.05 mg/Kg	Intraoperative: NA	Tumor detection + Segmentation	\	NA	ACCEPT
Alomari *et al.*[153]	2023	Prospective study	50	Minimally Invasive Surgery	NA	\	Stryker	HCC, CRLM	NA	NA	HCC: 0.5 mg/kg, CRLM: 0.5 mg/kg or 2.5 mg body PS: 0.025-12.5 mg body NS: 0.025-25 mg body	PS and NS: intraoperative HCC: 7-14 days before surgery CRLM: 3-7 days before surgery or 24 hours	Tumor detection + Segmentation	\	\	HIGH
Cai *et al.*[154]	2023	Retrospective study	86	Open, Minimally Invasive Surgery	NA	\	Stryker	HCC, CRLM	54.7 ± 15.3(HCC) 55.7 ± 9.6 (CRLM)	46/52 (HCC) 1/34 (CRLM) = 54.6%	Preoperative: 0.5 mg/Kg Intraoperative:1.25 mg NS technique	Preoperative: 5 days before surgery (ICG R15<=7%) 7 day before surgery (ICG R15>7%); NS	Tumor detection + Segmentation	\	15.38	ACCEPT
Weixler *et al.*[155]	2023	Prospective study	66	Open, Minimally Invasive Surgery	NA	\	Spectrum	HCC, CRLM, CCA, others	65.5 (IQR 58.7–73.9)	3.03	Preoperative: 5 mg/ml, 2 ml containing 10 ml	Preoperative: 24 h before surgery; PS	Tumor detection + Segmentation	\	25.75	LOW
Tao *et al.*[156]	2023	Post hoc analysis	16	Minimally	NA	\	PINPOINT™	HCC	56.56 ± 9.49	50	0.05–0.10 mg/kg for	Preoperative: 24-	Tumor detectio	\	\	

		of a prospective study		Invasive Surgery			imaging system (NOVADAQ, Canada) or DPM-III-01 imaging system (DPM, China)				tumor imaging; 0.5 mg for anatomic resection-negative staining	72 h prior to surgery Intraoperative: NS	n + Segmentation			ACCEPT
Rocca *et al.*[14]	2024	Retrospective study	45	Minimally Invasive Surgery	\	Da Vinci, Xi System	NA	CRLM	66.53 ± 12.67	NA	0.25 mg/kg	24 h before/during surgery	Tumor detection + Segmentation	\	22	ACCEPT

Table S4. Protocols and applications of Indocyanine Green Fluorescence during liver transplant. Abbreviations: HCC, Hepatocellular Carcinoma; CCA, Cholangiocarcinoma; CRLM, Colorectal Liver Metastases; PLC, Primary Liver Cancer; FNH, Focal Nodular Hyperplasia; GIST, gastrointestinal stromal tumour, SCC, Squamous Cell Carcinoma; LT, Liver Transplant; ICG, Indocyanine Green Fluorescence; NIR, near-infrared camera; PDE, Photodynamic Eye (Hamamatsu Photonics Co., Shizuoka, Japan); PIN, PINPOINT (Stryker Co., Michigan, US); HEMS, HyperEye Medical System (Mizuho Co., Ltd., Tokyo, Japan); Visera, Visera Elite II (Olympus Co., Tokyo, Japan); Image 1/Image 1S, Image 1/Image1 S camera systems (KARL STORZ SE and Co. KG, Tuttlingen, Germany); STORZ, KARL STORZ SE and Co. KG (Tuttlingen, Germany); PS, Positive Staining; NS, Negative Staining; NA, Not Available.

Author	Year	Study type	Number of patients	Type of surgery (Open, Minimally Invasive Surgery)	Type of Laparoscopic System	Type of Robot System	Type of NIR	Disease (HCC, CCA, CRLM or others)	Age (mean, median ±SD or range)	Cirrhosis (%)	Dose of ICG (mg/kg, ml)	Timing of administration	Type of navigation (Tumor detection, Segmentation, both)	Other uses	Major hepatectomy (%)	SIGN
**Troisi *et al.*[157]**	2014	Case report	1	Minimally Invasive Surgery	30° optical device (Karl Storz ICG fluorescent laparoscope)	\	30° optical device (Karl Storz ICG fluorescent laparoscope)	Living donor hepatectomy for glycogen storage disease type IV	21	0	0.1 mg/kg	Intraoperative: NS	Identify biliary ducts	\	\	LOW
**Tomassini *et al.*[158]**	2015	Prospective study	11	Minimally Invasive Surgery	30° optical Storz ICG camera, Karl Storz Gmbh & Co.	\	30° optical Storz ICG camera, Karl Storz Gmbh & Co.	Living donor hepatectomy	NA	\	Protocol A: 2.5 mg i.v; Protocol B: 2.5 mg i.v; Protocol C: 1 mg i.v; Protocol D: 2.5 mg i.d; Protocol E: 5 mg i.d	Immediate (Protocol A-D-E) 60-90 min before bile duct resection (Protocol B-C): NA	Segmentation	\	\	LOW
**Panaro *et al.*[159]**	2018	Retrospective study	6 livers	Open	\	\	VITOM®, KARL STORZ GmhH & Co. KG, Tuttlingen, Germania	NA	NA	100	0.5 mg/kg	Intraoperative	To assess the graft bile duct perfusion in order to identify the appropriate area of duct transection before performing	\	\	ACCEPT

													the anastomosis			
**Kim *et al.*[160]**	2021	Retrospective study	46	Open	\	\	PIN, others	Healthy LT	53.7 ± 11.8	0	0.025 mg/kg	Intraoperative: NS	Segmentation	\	100	ACCEPT

Table S5. Protocols and applications of Indocyanine Green Fluorescence to bile leak identifications. Abbreviations: HCC, Hepatocellular Carcinoma; CCA, Cholangiocarcinoma; CRLM, Colorectal Liver Metastases; PLC, Primary Liver Cancer; FNH, Focal Nodular Hyperplasia; GIST, gastrointestinal stromal tumour, SCC, Squamous Cell Carcinoma; LT, Liver Transplant; ICG, Indocyanine Green Fluorescence; NIR, near-infrared camera; PDE, Photodynamic Eye (Hamamatsu Photonics Co., Shizuoka, Japan); PIN, PINPOINT (Stryker Co., Michigan, US); HEMS, HyperEye Medical System (Mizuho Co., Ltd., Tokyo, Japan); Visera, Visera Elite II (Olympus Co., Tokyo, Japan); Image 1/Image 1S, Image 1/Image1 S camera systems (KARL STORZ SE and Co. KG, Tuttlingen, Germany); STORZ, KARL STORZ SE and Co. KG (Tuttlingen, Germany); PS, Positive Staining; NS, Negative Staining; NA, Not Available.

**Author**	**Year**	**Study type**	**Number of patients**	**Type of surgery (Open, Minimally Invasive Surgery)**	**Type of Laparoscopic System**	**Type of Robot System**	**Type of NIR**	**Disease (HCC, CCA, CRLM or others)**	**Age (mean, median ±SD or range)**	**Cirrhosis (%)**	**Dose of ICG (mg/kg, ml)**	**Timing of administration**	**Type of navigation (Tumor detection, Segmentation, both)**	**Other uses**	**Major hepatectomy (%)**	**SIGN**
Sakaguchi *et al.*[161]	2010	Cohort studies	27	Open	\	\	PDE	HCC, CRLM, others	65.7 ± 10.8	33.3	0.05 mg/ml	Intraoperative	Bile leak	\	\	ACCEPT
Kaibori *et al.*[162]	2011	Randomized Clinical Trial	102 (50 ICG control group; 52 ICG PDE group)	Open	\	\	Hamamatsu Photonics K.K.	HCC, CCA, CRLM, others	ICG Control group: 68.2 (11.0) ICG PDE group: 70.4 (5.5)	33.3	10 ml of dilute ICG solution 2.5 mg/ml	Intraoperative	Bile leak	\	Extended hemihepatectomy= 10.8	HIGH
Umemura *et al.*[163]	2021	Case report	1	Minimally Invasive Surgery	NA	\	PIN	Liver cysts	NA	NA	2.5 mg/mL	Intraoperative put into the biliary tract via endoscopic nasal biliary drainage: NA	Bile leak	\	0	LOW
Hanaki *et al.*[164]	2023	Prospective study	40	Open, Minimally Invasive Surgery	NA	\	AIM	HCC, Metastases, benign lesion	NA	NA	Intraoperative: 10 mg/ml	Preoperative: 1-2 hours before hepatic dissention	Bile leak	\	NA	ACCEPT

Sasaki *et al.[165]*	2023	Randomized Clinical Trial	3	Open, Minimally Invasive Surgery	NA	\	VISERA ELITE II, IMAGE1 S™ 4U RUBINA (KARL STORZ, Tuttlingen, Germany), Stryker	HCC, CRLM. others	71.66 (64, 77, 74)	0	Case one: 0.01 mg/ml; Case two: 0.05 mg/ml; Case three: 0.025 mg/ml	Intraoperative	Bile leak	\	\	ACCEPT

Table S6. Protocols and applications of Indocyanine Green Fluorescence in particular applications. Abbreviations: HCC, Hepatocellular Carcinoma; CCA, Cholangiocarcinoma; CRLM, Colorectal Liver Metastases; PLC, Primary Liver Cancer; FNH, Focal Nodular Hyperplasia; GIST, gastrointestinal stromal tumour, SCC, Squamous Cell Carcinoma; LT, Liver Transplant; ICG, Indocyanine Green Fluorescence; NIR, near-infrared camera; PDE, Photodynamic Eye (Hamamatsu Photonics Co., Shizuoka, Japan); PIN, PINPOINT (Stryker Co., Michigan, US); HEMS, HyperEye Medical System (Mizuho Co., Ltd., Tokyo, Japan); Visera, Visera Elite II (Olympus Co., Tokyo, Japan); Image 1/Image 1S, Image 1/Image1 S camera systems (KARL STORZ SE and Co. KG, Tuttlingen, Germany); STORZ, KARL STORZ SE and Co. KG (Tuttlingen, Germany); PS, Positive Staining; NS, Negative Staining; NA, Not Available.

Author	Year	Study type	Number of patients	Type of surgery (Open, Minimally Invasive Surgery)	Type of Laparoscopic System	Type of Robot System	Type of NIR	Disease (HCC, CCA, CRLM or others)	Age (mean, median ±SD or range)	BMI (mean, median ±SD or range)	Cirrhosis (%)	Dose of ICG (mg/kg, ml)	Timing of administration	Type of navigation (Tumor detection, Segmentation, both)	Other uses	Major hepatectomy (%)	SIGN
Kawaguchi *et al.[166]*	2015	Retrospective study	24	Minimally Invasive Surgery	NA	\	Olympus Medical Systems	CRLM	67.1 (26.3-79.4)	26.4 (18.1-34.9)	NA	1 mL of ICG (0.025 mg/mL of Infracyanine; Laboratoire Serb	After intubation in the operative room	Bile duct visualization	\	Major laparoscopic liver resection: 54.2	LOW
Tanaka *et al.[167]*	2020	Case report	1	Minimally Invasive Surgery	NA	\	NA	HCC	69	NA	NA	9.5 mL of mixture A (5 mL ICG and 5 mL Lipiodol®) and mixture B (5 mL ICG and Gelpart® [Gelpart 1 mm 80 mg; Nippon Kayaku, Tokyo, Japan] mixed in 5 mL of contrast medium)	Preoperative	Embolization	\	\	LOW
Ruzzenente *et al.[168]*	2022	Prospective study	18	Open, Minimally Invasiv	NA	\	Stryker™	HCC, CRLM, CCA, others	71 (58-78)	NA	33.3	1 ml of 25 mg/20 ml	Intraoperative	Lymph node sentinel	\	50	ACCEPT

e<br>Surgery
